# Myocardial blood flow quantification by Rb-82 cardiac PET/CT: A detailed reproducibility study between two semi-automatic analysis programs

**DOI:** 10.1007/s12350-015-0151-2

**Published:** 2015-05-21

**Authors:** Vincent Dunet, Ran Klein, Gilles Allenbach, Jennifer Renaud, Robert A. deKemp, John O. Prior

**Affiliations:** Department of Nuclear Medicine and Molecular Imaging, Lausanne University Hospital, Rue du Bugnon 46, 1011 Lausanne, Switzerland; University of Ottawa Heart Institute, Cardiac PET Centre, Ottawa, Canada

**Keywords:** Software, PET, rubidium-82, quantification, myocardial perfusion, concordance, accuracy, precision, agreement, comparison

## Abstract

**Background:**

Several analysis software packages for myocardial blood flow (MBF) quantification from cardiac PET studies exist, but they have not been compared using concordance analysis, which can characterize precision and bias separately. Reproducible measurements are needed for quantification to fully develop its clinical potential.

**Methods:**

Fifty-one patients underwent dynamic Rb-82 PET at rest and during adenosine stress. Data were processed with PMOD and FlowQuant (Lortie model). MBF and myocardial flow reserve (MFR) polar maps were quantified and analyzed using a 17-segment model. Comparisons used Pearson’s correlation *ρ* (measuring precision), Bland and Altman limit-of-agreement and Lin’s concordance correlation ρ_c_ = ρ·*C*_b_ (*C*_b_ measuring systematic bias).

**Results:**

Lin’s concordance and Pearson’s correlation values were very similar, suggesting no systematic bias between software packages with an excellent precision ρ for MBF (ρ = 0.97, ρ_c_ = 0.96, *C*_b_ = 0.99) and good precision for MFR (ρ = 0.83, ρ_c_ = 0.76, *C*_b_ = 0.92). On a per-segment basis, no mean bias was observed on Bland-Altman plots, although PMOD provided slightly higher values than FlowQuant at higher MBF and MFR values (*P* < .0001).

**Conclusions:**

Concordance between software packages was excellent for MBF and MFR, despite higher values by PMOD at higher MBF values. Both software packages can be used interchangeably for quantification in daily practice of Rb-82 cardiac PET.

## Introduction

Myocardial perfusion imaging is an important step for diagnosis and management of coronary artery disease. Cardiac positron emission tomography (PET) with various positron-emitting tracers such as ^13^N-Ammonia, ^15^O-water, or the cationic potassium analog ^82^Rb, is a well-known modality to study myocardial perfusion at rest and in response to physiological or pharmacological stress.^1^,^2^ Moreover, it allows regional myocardial blood flow (MBF) quantification and assessment of myocardial flow reserve (MFR), extending the diagnostic potential of standard myocardial perfusion imaging, especially for patients with 3-vessel disease, bundle branch block.^3^ Flow measurements are useful for the assessment of the extent and severity of coronary epicardial vascular disease, as well as diffuse abnormal microcirculatory function without coronary stenosis.^4^ Furthermore, it leads to a better statement of the coronary risk, screening of predictive factors of cardiovascular events, and monitoring of the effectiveness of therapeutic strategies for cardiovascular risk reduction.^5^-^7^

MBF can be estimated by automatic or semi-automatic software packages, following a kinetic modeling approach, using time-activity curves derived from dynamic PET acquisitions. The choice of the compartmental modeling method depends on the biodistribution and kinetics of the perfusion radiotracer. With the increased availability of PET scanners mainly driven by oncological applications, there is a clear interest in the use of cyclotron-free, generator-produced radioisotope ^82^Rb.^8^ Several tracer kinetic models have been validated for MBF quantification by ^82^Rb cardiac PET.^9^-^13^ In particular, the 1-tissue-compartment model (also called 2-compartment model with a vascular and a cellular compartment) described by Lortie et al. allows MBF estimation with good test-retest repeatability and reproducibility among centers.^13^-^17^ A recent review of 10 different software packages for MBF quantification has been performed by Saraste et al. and shows that MBF quantification is advancing to become a clinical reality.^18^ However, Bateman and Case questioned in a recent editorial as to whether MBF values were sufficiently robust for altering clinical management.^19^ Thus, before answering this question, there is a need to better understand the inherent differences between software packages.

**Figure 1:**
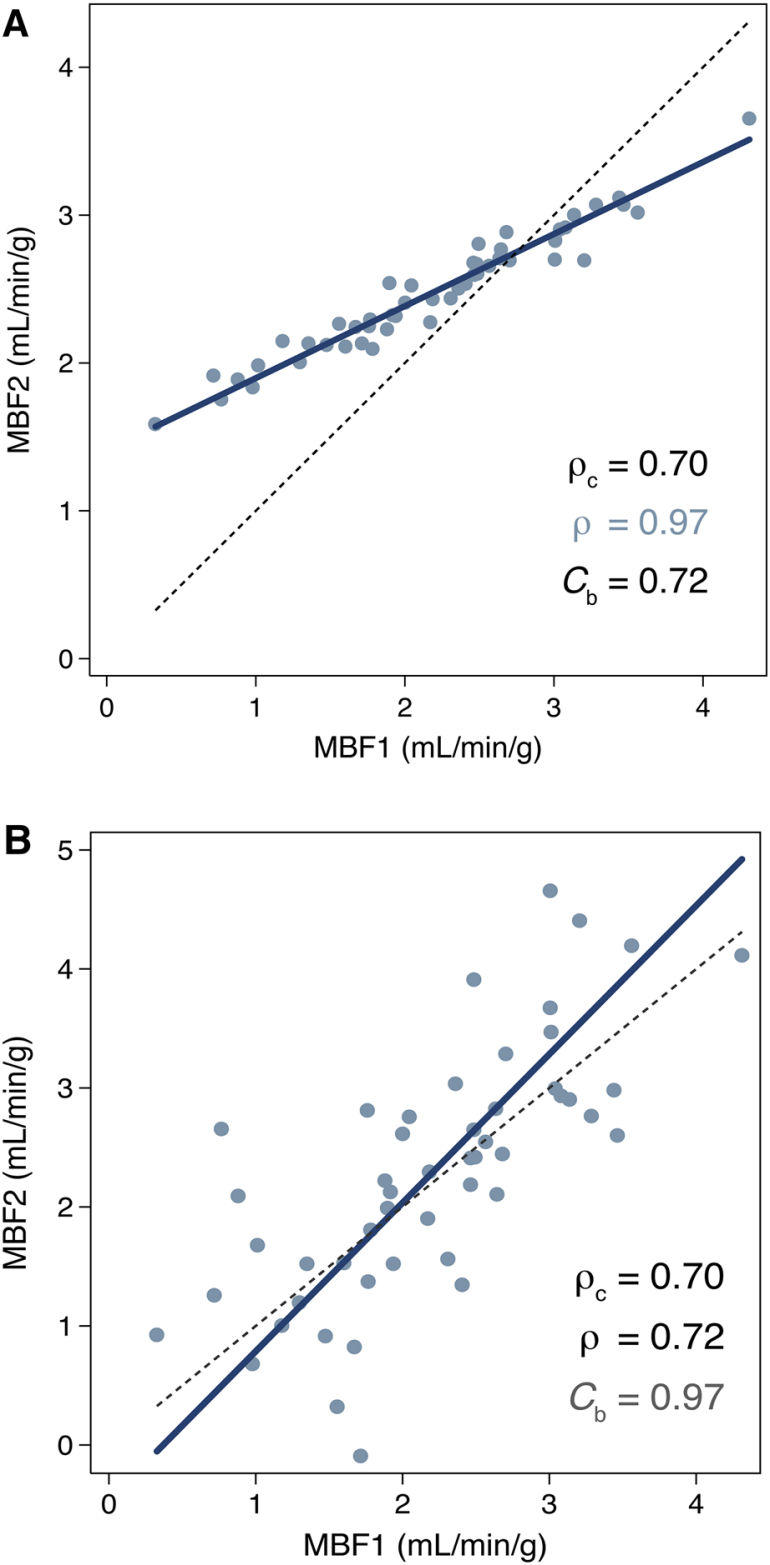
Illustration of the Lin’s concordance with separate assessment of (**A**) of how far the fitted relationship between *x* and *y* data deviates from the 45° concordance line through the origin (systematic bias) and (**B**) how far each data point deviates from the fitted line (precision = Pearson’s ρ). The identity line (*dashed*, *y* = *x*) and the reduced major axis linear regression (*solid*) are shown. Both graphics illustrate identical concordance ρ_c_ = 0.70. However, (**A**) shows an excellent precision (ρ = 0.97) with low scatter but a systematic bias (*C*_b_ = 0.72), while (**B**) shows no bias (excellent *C*_b_ = 0.97) and a lower precision with more scattered measurements (fair ρ = 0.72)

To the best of our knowledge, several studies compared the effects of different acquisition protocols, modeling approaches, and software packages on MBF quantitative analysis,^20^-^24^ but none has compared the effect on quantifying ^82^Rb myocardial blood flow with different software packages and its clinical consequences using contemporary statistical methods, such as Lin’s analysis of concordance.^25^,^26^ This method is analogous to the kappa coefficient, but for continuous scale data. It was developed more than 20 years ago, but it is not so frequently used outside statistical journals. It avoids many drawbacks of conventional comparison methods used traditionally (mean difference, Pearson’s correlation, linear regression, or intra-class correlation). It has rarely been used in imaging and only once in relation to comparing PET-measured MBF.^13^ Thus, we aimed to understand the differences in MBF quantification between two available processing software packages for ^82^Rb cardiac PET studies using Lin’s analysis of concordance. This statistic of agreement provides separate measurements of precision (agreement of individual measurements or the degree of scatter) and systematic bias (agreement of the mean measurements) of MBF quantification (Figure 1), whose understanding is needed before adopting widespread clinical use.

## Materials and Methods

### Population

Fifty-one patients (20 women, 31 men) with known or suspected coronary artery disease undergoing myocardial blood flow imaging at the Lausanne University Hospital participated in the study. The population clinical characteristics are summarized in Table 1. Each patient underwent dynamic ^82^Rb cardiac PET acquisition at rest and during pharmacological stress by infusion of adenosine (140μg/kg/min over 6 minutes) with qualitative perfusion analysis, and MBF and MFR assessment. The local Ethics Committee approved the study protocol and all subjects gave written informed consent prior to enrolment.

**Table 1:** Population clinical characteristics (*n* = 51)

	Mean ± SD or # (%)
Age (years)	63 ± 12
Gender	31 (61%) M; 20 (39%) W
Weight (kg)	81 ± 17
Body mass index (kg/m^2^)	28.2 ± 5.4
Obesity: body mass index >30 kg/m^2^	35 (69%)
Diabetes history	16 (31%)
Arterial hypertension: ≥140/90 mmHg	30 (59%)
Dyslipidemia: LDL ≥ 4.1 mmol/L (160 mg/dL) or HDL ≤ 0.8 mmol/L (30 mg/dL)	23 (45%)
Smoking history	16 (31%)
Family history of heart disease	8 (16%)
Known coronary artery stenosis	7 (14%)
Previous coronary artery bypass surgery	5 (10%)

### Patient Preparation

Patients were instructed to refrain from caffeine-containing beverages for at least 12 hours and from food at least 6 hours prior to the test. An EKG at rest was recorded to ensure the absence of an II- or III-degree atrio-ventricular block that is a contraindication for adenosine. Afterwards, a venous catheter was placed on one forearm, and an armband for blood pressure monitoring was placed on the other arm. Every patient was imaged in supine position with arms above the head, wedged with cushions to prevent sliding. Blood pressure was controlled at rest before any infusion of adenosine to ensure the absence of hypotension.

### Acquisition Protocol

Data acquisitions were performed with a Discovery LS PET/CT (GE HealthCare, Waukesha, WI) in 2-D mode using a multi-frame acquisition protocol over 6 minutes (12 × 8 seconds, 5 × 12 seconds, 1 × 30 seconds, 1 × 60 seconds, 1 × 120 seconds) started immediately after a 30-seconds square-wave infusion of 1450 MBq of [^82^Rb] rubidium chloride at rest.^27^ The same acquisition protocol was used for stress imaging starting 2 minutes after the beginning of adenosine infusion. Two low-dose CT scans (120 keV, 10 mAs) were acquired for attenuation correction, one just before the rest study and one immediately after the stress study. CT images were manually reviewed for accurate coregistration with the PET images. Dynamic transverse images were reconstructed using OSEM with a Hann loop filter of 2.34 mm full-width at half maximum (FWHM) and a Hann post-filter of 3.27 mm FWHM. The arterial blood pressure and heart rate, as well as the 12-lead EKG were continuously monitored. The total time in the PET/CT scanner was about 20 minutes and the effective dose due to ^82^Rb was about 2 mSv for both rest and stress acquisitions, including the CT dose.^28^

### Image Processing

Datasets were systematically processed with two software packages for quantitative analysis of MBF: (i) the commercial PMOD 3.0 (PMOD Technologies Ltd. Zurich, Switzerland) and (ii) the academic FlowQuant 2.1.3.^14^,^17^ Both programs use the same, previously described 1-tissue-compartment model, corrected for ^82^Rb flow-dependent extraction, myocardial partial-volume recovery, and blood spillover.^14^ Note that both software packages correct for spillover of the left ventricle (LV) blood, while PMOD also corrects for the right ventricle blood spillover in septal segments.

The myocardial uptake *C*_m_(*t*) was determined by averaging the late image frames and performing sampling as described below for each software package. PMOD used the early phase to determine the LV blood pool region, while FlowQuant used a region extending from mid cavity to atrium at maximum distance from the mid-myocardium region. *C*_LV_(*t*), assumed to be the uptake function of the model, was acquired by sampling in the respective blood pool regions. The model parameters (*K*_1_, *k*_2_, *F*_LV_) were estimated using a weighted least-squares method to satisfy Equations (1) and (2):

1 $$ C_{\text{m}} \left( t \right) = K_{1} \cdot e^{{{-}k2 \cdot t}} \otimes C_{\text{LV}} \left( t \right), $$

2 $$ C_{\text{PET}} \left( t \right) \, = F_{\text{LV}} \cdot C_{\text{LV}} \left( t \right) \, + \, \left( {1 \, {-}F_{\text{LV}} } \right)\, \cdot \,C_{\text{m}} \left( t \right), $$

where *F*_LV_ is a real number between 0 and 1 and (1 − *F*_LV_) is a regional estimation of the myocardial partial-volume recovery coefficient. The *K*_1_ uptake parameter was related to MBF using a Renkin-Crone function:

3 $$ K_{1} = MBF\, \cdot \,\,\left( {1 \, {-}a\, \cdot \,e^{{{-}b/{\text{MBF}}}} } \right). $$

For both software packages, the *a* and *b* constant values were identical, respectively, *a* = 0.77 and *b* = 0.63 mL/min/g.^14^

#### PMOD processing

After loading DICOM files, PMOD automatically generated blood pool (BP) and myocardial images from dynamic uptake series by averaging respective frames, from 10 to 70 seconds for BP and from 2 to 6 minutes for myocardium, then smoothing with a 3-D Gaussian filter of 6-mm FWHM. Then, standard reorientation of the heart was performed; PMOD determined volumes-of-interest (VOI) in the left ventricle, right ventricle, and in the centerline of myocardium were applied before sampling and the corresponding time-activity-curves (TACs) were generated. Finally, uptake parameters were determined by fitting the tracer kinetic model to the TACs (Equation 1, 2), leading to MBF calculation (Equation 3), except that for septal segments Equation 2 was replaced by

4 $$ C_{\text{PET}} \left( t \right) \, = \, F_{\text{LV}} \, \cdot \,C_{\text{LV}} \left( t \right) \, + F_{\text{RV}} \, \cdot \,C_{\text{RV}} \left( t \right) \, + \, \left( { 1 { }{-}F_{\text{LV}} {-}F_{\text{RV}} } \right)\, \cdot \,C_{\text{m}} \left( t \right), $$

where *F*_LV_ and *F*_RV_ are real numbers between 0 and 1 and (1 − *F*_LV_ − *F*_RV_) is a regional estimation of the myocardial partial-volume recovery coefficient. The resulting parameters from each segment were finally displayed in a polar plot corresponding to the 17-segment AHA scheme, and used for generating MBF reports.

#### FlowQuant processing

After loading adequate DICOM files, FlowQuant proceeded to perform standard reorientation of the heart using a myocardium uptake image generated by summing the last 5 uptake frames (2.2-6 minutes) and applying a 3-D Gaussian filter (complementing the image resolution to 12-mm FWHM). After reorientation, the software detected the mid-myocardium in the image volume to generate myocardial time-activity-curves. The BP median TAC was created from three samples located in the LV cavity, the LV base, and the left atrium. The 1-tissue-compartment model was then fitted to the measured TACs and uptake rates were determined, and subsequently converted to MBF estimates. FlowQuant finally generated a series of polar maps for the activity uptake, *MBF*, *K*_1_, blood spillover (*F*_LV_), as well as the reduced χ^2^ and *R-squared* values of the fit. Results of MBF at rest, at stress and MFR were compared for global LV and segmental territories using the 17-segment AHA myocardial model. For all the patients VOI positions were checked to be similar when processing data with both software packages to ensure quality of the comparison.

### Statistical Analysis

Values are presented as mean ± SD. Continuous mean values were compared with a Student’s *t* test. Relations between PMOD and FlowQuant results were assessed using Pearson’s correlation (indicating precision), ρ, Bland-Altman limit-of-agreement (LOA), and Lin’s concordance correlation coefficient, ρ_c_, a measure of both precision and bias.^25^,^26^ In fact, Lin’s concordance correlation is the most appropriate test to measure equivalence of two measurement methods, and ranges from +1 (perfect agreement) to 0 (no agreement). In Lin’s formalism, the *precision* (Pearson’s correlation ρ) illustrates the agreement of the individual measurements from the best-fit line and the *trueness* (defined by the bias correction factor *C*_b_ = ρ_*c*_/ρ) indicates the agreement of the mean test results from the 45° line-of-identity through the origin (see Figure 1). The analysis of concordance avoids drawbacks of some other conventional comparison methods such as the Pearson’s ρ used alone (which fails to detect a departure from the 45° line through the origin), the paired t-test (which could reject a reproducible method due to small residual error among means), the least-square approach (slope = 1, intercept = 0, which would fail to detect departure from the best-fit line if data are highly scattered), and the coefficient of variation or the intra-class correlation coefficient (which do not distinguish bias from imprecision).^26^ The values of ρ and ρ_c_ can be characterized using the Landis and Koch scale (0.2-0.4: fair; 0.4-0.6: moderate; 0.6-0.8: substantial; 0.8-1.0 almost perfect).^29^ The reduced major axis linear regression (line going through the intersection of the means with a slope given by the sign of the Pearson’s correlation and the ratio of the respective standard deviations) and the locally weighted regression curves were used in the graphical representations. A *P* value < .05 was considered statistically significant. All tests were performed using Stata 10.1 statistical analysis software (Stata Corporation, College Station, TX, USA).

## Results

### Process Quality Criteria

All 102 studies were successfully processed with both FlowQuant and PMOD programs. The reorientation phase was completed automatically in all cases. For the myocardial segmentation phase, while FlowQuant ran automatically for 94% of the stress + rest studies (3 failures in 102 studies), PMOD failed more frequently (in about 30% of the cases) requiring manual definition of the myocardial VOI. For three patients, the automatic left ventricle VOI delineation did not define blood pool regions at the same position at stress. These three patients were excluded from the statistical analysis and images were reviewed to obtain similar VOI position with the two software packages. These three cases are discussed separately below. Both programs proceeded successfully through automatic myocardial VOI sampling and kinetic modeling.

### LV Quantitative Results

**Figure 2:**
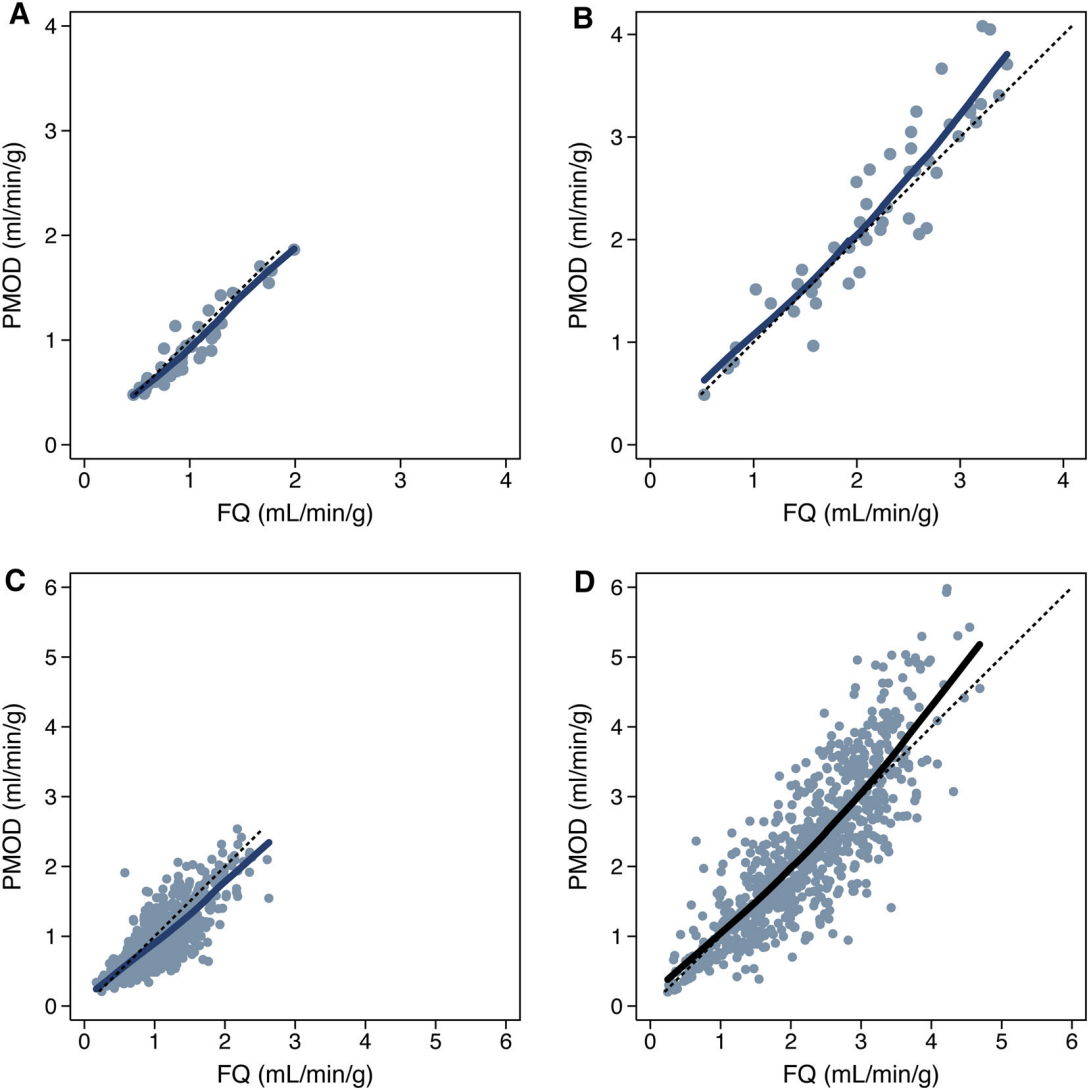
Comparison of MBF values according to software package. Comparison of global* left ventricular* MBF values for (**A**) rest and (**B**) stress flows. Comparison at the 17-segment level with MBF values for (**C**) rest and (**D**) stress flows. The identity line (*dashed*, *y* = *x*) and the locally weighted regression curve (*solid*) are presented

**Figure 3:**
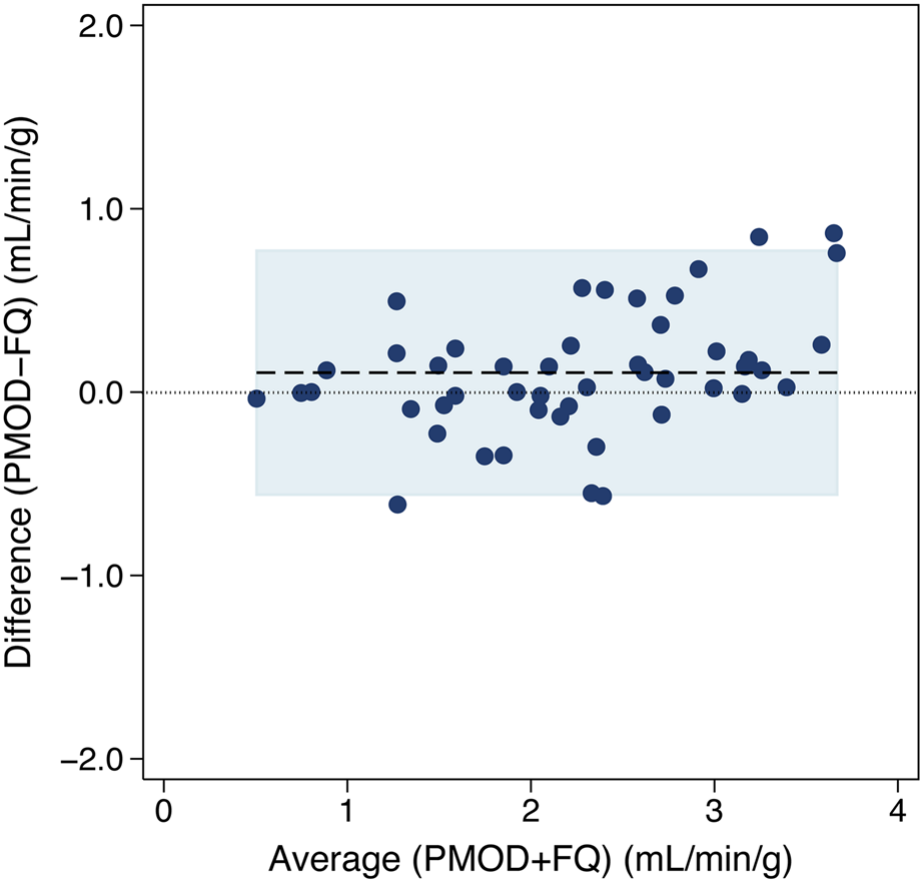
Bland-Altman plot of difference vs average global LV stress MBF (n = 48) between software packages. Legend: *dashed line* = mean difference = 0.11 mL/min/g [5%, *P* = .036]; *shaded* *area* = ±95% limits of agreements [−0.56-0.77 mL/min/g]

MBF at rest and stress, as well as MFR for the global LV (n = 48) and for the 17 segments (n = 816) are displayed in Figure 2; *mean* *±* *SD* are given in Table 2. At rest, there was a small (−7%), but statistically significant mean difference between mean global LV MBF using PMOD vs FlowQuant (−0.07 ± 0.11 mL/min/g, *P* = .0001). Pearson’s correlation (ρ = 0.95) and Lin’s concordance (ρ_c_ = 0.93) were similar, also indicating that no systematic bias was present (*C*_b_ = 0.98) between software packages. At stress, there was also a small but not clinically significant (5%) mean difference (0.11 ± 0.34 mL/min/g, *P* = .036) in global LV MBF with PMOD vs FlowQuant, leading to a small (12%) difference in MFR (0.28 ± 0.45, *P* = .0001). Corresponding Pearson’s correlation and Lin’s concordance were also similar to each other (ρ = 0.93, ρ_c_ = 0.91), indicating no systematic bias for MBF (*C*_b_ = 0.98) and MFR (ρ = 0.83, ρ_c_ = 0.76, *C*_b_ = 0.92). The limits of agreement of the Bland-Altman plot of global LV stress MBF were also fairly narrow (30%) as illustrated in Figure 3.

**Table 2:** Global LV MBF, 17-segment MBF, and MFR results for both PMOD and FlowQuant software packages

Variable	n	PMOD	FlowQuant	*P*
Rest global LV MBF (mL/min/g)	48	0.94 ± 0.35	1.01 ± 0.35	0.0001
Stress global LV MBF (mL/min/g)	48	2.30 ± 0.89	2.20 ± 0.76	0.04
Stress + Rest Global LV MBF (mL/min/g)	96	1.60 ± 0.84	1.62 ± 0.96	0.5
Global LV MFR	48	2.52 ± 0.81	2.24 ± 0.67	0.0001
Rest 17-segment MBF (mL/min/g)	816	0.92 ± 0.42	1.01 ± 0.42	<0.0001
Stress 17-segment MBF (mL/min/g)	816	2.25 ± 1.07	2.23 ± 0.87	0.4
Stress + Rest 17-segment MBF (mL/min/g)	1632	1.58 ± 1.53	1.62 ± 0.92	0.0004
17-segment MFR	816	2.63 ± 1.29	2.36 ± 1.03	<0.0001

Values displayed as mean ± SD. *LV*, Left ventricle; *MBF*, myocardial blood flow; *MFR*, myocardial flow reserve

**Figure 4:**
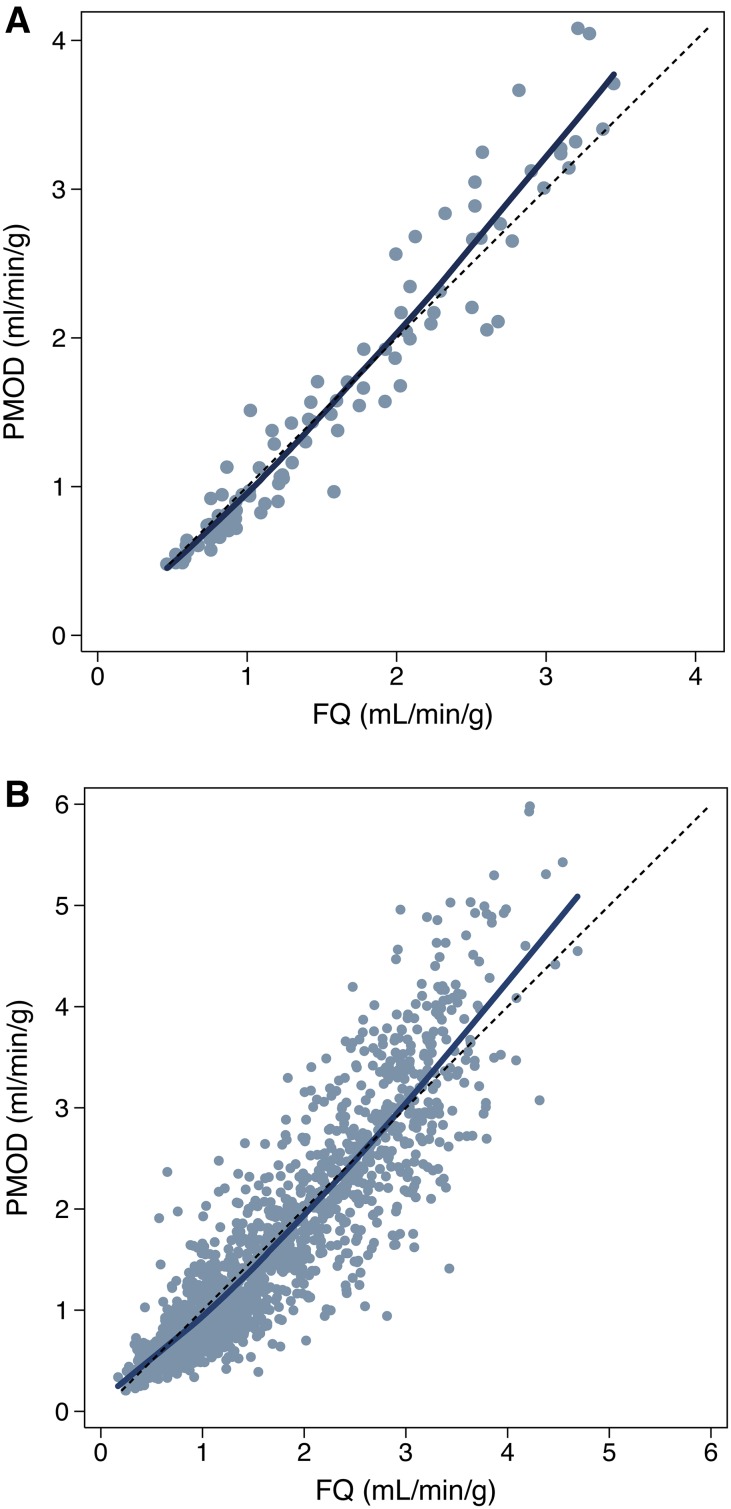
Comparison of MBF values according to software package: (**A**) comparison of global LV MBF values; (**B**) comparison of regional 17-segment MBF values. The identity line (*short dash*, *y* = *x*) and the locally weighted regression curve (*solid*) are presented

At the 17-segment level, there was also good agreement in MBF results using PMOD vs FlowQuant. Small, but statistically significant mean differences were found at rest (−0.10 ± 0.26 mL/min/g, *P* < .001) and for MFR (0.27 ± 0.89, *P* < .0001), but not at stress (0.02 ± 0.57 mL/min/g, *P* = .39) (Table 2). Pearson’s correlation and Lin’s concordance were still good for rest (ρ = 0.81, ρ_c_ = 0.79, *C*_b_ = 0.97) and stress (ρ = 0.84, ρ_c_ = 0.83, *C*_b_ = 0.98) and for MFR (ρ = 0.73, ρ_c_ = 0.69, *C*_b_ = 0.95) (Figure 4).

As there was no spillover correction for the right ventricle blood in FlowQuant compared to PMOD, we wanted to compare septal segments (2, 3, 8, 9, and 14, with right ventricle spillover correction in PMOD) to non-septal segments (1, 4-7, 10-13, and 15-17). However, there was no difference in Pearson’s correlation and Lin’s concordance (septal segments: ρ = 0.91, ρ_c_ = 0.89, *C*_b_ = 0.98 vs non-septal segments: ρ = 0.90, ρ_c_ = 0.90, *C*_b_ = 0.99, *P* > .05).

To assess whether MBF results were influenced by the presence of regional perfusion heterogeneity, as often seen with ischemia or infarction, two groups of patients were determined by consensus of two experienced physicians according to the presence (n = 20) or the absence (n = 28) of perfusion defect on the rest/stress uptake images. There was no difference in global rest LV MBF results between subgroups using either software (*P* > .27), but there was a statistically significant difference between programs (*P* < .01) for both subgroups (Table 3). There was no difference between PMOD and FlowQuant estimated global stress MBF in the subgroup with perfusion heterogeneity (*P* = .16) nor in the subgroup without (*P* = .13) perfusion heterogeneity (Table 3). Global stress LV MBF was significantly different between patients with and without perfusion heterogeneity using either PMOD (*P* = .036) or FlowQuant (*P* = .009). MFR was significantly lower for patients with perfusion heterogeneity than those with perfusion homogeneity when assessed by FlowQuant (*P* = .022) but not by PMOD (*P* = .2).

**Table 3:** Software comparison in patients with or without regional perfusion heterogeneities

Perfusion	PMOD		FlowQuant	
	Heterogeneous n = 20	Homogenous n = 28	Heterogeneous n = 20	Homogenous n = 28
Rest global LV MBF (mL/min/g)	0.87 ± 0.31	0.98 ± 0.37	0.94 ± 0.32*	1.08 ± 0.37*
Stress global LV MBF (mL/min/g)	1.98 ± 0.92	2.53 ± 0.82^†^	1.87 ± 0.78	2.43 ± 0.65^†^
Global LV MFR (unitless ratio)	2.35 ± 1.01	2.65 ± 0.63	1.98 ± 0.66^‡^	2.43 ± 0.61*^,§,||^

*PMOD vs FlowQuant in heterogeneous (*P* = .002) and homogeneous perfusion (*P* = .01); ^†^Heterogeneous vs homogeneous perfusion with PMOD (*P* = .04) and FlowQuant (*P* = .009); ^‡^PMOD vs FlowQuant in heterogeneous perfusion (*P* = .003); ^§^PMOD vs FlowQuant in homogeneous perfusion (*P* = 0.01); ^||^Heterogeneous vs homogeneous perfusion with FlowQuant (*P* = 0.02). *LV*, Left ventricle; *MBF*, myocardial blood flow; *MFR*, myocardial flow reserve

When both subgroups were compared based on the 17-segment MBF values, a statistical difference between stress MBF results was observed with both programs (PMOD: *P* < .0001; FlowQuant: *P* < .0001). There was also a significant difference between subgroups at rest (PMOD: *P* = .0002; FlowQuant: *P* = .0005). In both subgroups, stress MBF was not statistically different between PMOD and FlowQuant (*P* > .45), but MFR was significantly higher with PMOD than FlowQuant (*P* < .0001). MFR was significantly higher in patients without perfusion defect when assessed by FlowQuant (*P* < .0001) or PMOD (*P* = .0009). Pearson’s correlation and Lin’s concordance were very good for global LV MBF and 17-segment pooled MBF with perfusion heterogeneity (ρ = 0.95, ρ_c_ = 0.94, *C*_b_ = 0.99 and ρ = 0.89, ρ_c_ = 0.88, *C*_b_ = 0.99) or without (ρ = 0.97, ρ_c_ = 0.96, *C*_b_ = 0.99 and ρ = 0.91, ρ_c_ = 0.90, *C*_b_ = 0.99).

Finally, when pooling rest and stress global LV MBF (n = 96) or 17-segments values (n = 1632), PMOD showed slightly higher values than FlowQuant at the higher range of myocardial blood flows, as assessed by Bland-Altman and locally weighted regression analysis. The mean difference between software packages was therefore better for global LV MBF < 2.0 mL/min/g than for global LV MBF ≥ 2.0 mL/min/g (−6% ± 15% vs 7% ± 13%, *P* = .0001, n = 96). Comparing all the segments, there was a relative overestimation by PMOD software for the highest MBF ≥ 2.0 (mean difference 0.22 ± 0.57 mL/min/g, *P* < .0001, n = 475), which may lead to an overestimation of MFR (difference 0.5 ± 1.0, *P* < .0001). The mean difference between software packages was therefore better for MBF < 2.0 than for MBF ≥ 2.0 (−14% ± 31% vs 8% ± 20%, *P* < .0001, n = 1632). In spite of this finding, correlation and concordance between both software packages were very good for global LV MBF as well as for 17-segment values (ρ = 0.97, ρ_c_ = 0.96, *C*_b_ = 0.99 and ρ = 0.9, ρ_c_ = 0.9, *C*_b_ = 0.99, respectively) (Table 4).

**Table 4:** PMOD vs FlowQuant concordance correlation coefficients

	n	Pearson’s ρ [95% CI]	ρ_c_ [95% CI]	*C*_b_	Reduced major axis PMOD = *f* (FQ)
Global left ventricle					
Rest MBF (mL/min/g)	48	0.95 [0.91–0.97]	0.93 [0.89–0.97]	0.98	*y* = 0.99*x* − 0.06
Stress MBF (mL/min/g)	48	0.93 [0.88–0.96]	0.91 [0.86–0.95]	0.98	*y* = 1.18*x* − 0.29
Stress + Rest MBF (mL/min/g)	96	0.97 [0.95–0.98]	0.96 [0.94–0.97]	0.99	*y* = 1.15*x* − 0.22
MFR (unitless ratio)	48	0.83 [0.72–0.90]	0.76 [0.66–0.87]	0.92	*y* = 1.22*x* − 0.20
17-Segment level					
Rest MBF (mL/min/g)	816	0.81 [0.78–0.83]	0.79 [0.76–0.81]	0.97	*y* = 1.00*x* − 0.10
Stress MBF (mL/min/g)	816	0.84 [0.82–0.86]	0.83 [0.81–0.85]	0.98	*y* = 1.23*x* − 0.49
Stress + Rest MBF (mL/min/g)	1632	0.90 [0.90–0.91]	0.90 [0.89–0.90]	0.99	*y* = 1.15*x* − 0.28
MFR (unitless ratio)	816	0.73 [0.70–0.76]	0.69 [0.66–0.73]	0.95	*y* = 1.25*x* − 0.33

*ρ*, Pearson’s correlation (*precision*); *ρ*_c_, Lin’s concordance correlation; *C*_*b*_, *ρ*_c_/*ρ* = bias correction factor (*trueness*); *LV*, left ventricle; *MBF*, myocardial blood flow; *MFR*, myocardial flow reserve

When analyzing the proportion of normal vs abnormal studies, the results were very close, either at the patient level or at the segment level (Table 5). There was a small, but statistically significant difference at the segmental level that is not likely to be clinically relevant, as demonstrated by no significant difference at the whole left-ventricle level, or when using both stress MBF and MFR to define normal values (stress MBF ≥ 2.0 mL/min/g or MFR ≥ 2.0).

**Table 5:** PMOD vs FlowQuant classification of normal vs abnormal stress myocardial blood flow (MBF) and myocardial flow reserve (MFR)

Normal MBF or MFR values	n	PMOD	FlowQuant	Difference	*P*
Whole left-ventricle					
Stress MBF ≥ 2.0 mL/min/g	48	30 (63%)	31 (65%)	1 (2.1%)	0.84
MFR ≥ 2 (unitless ratio)	48	34 (71%)	30 (63%)	4 (8.3%)	0.40
17-Segment level					
Stress MBF ≥ 2.0 mL/min/g	816	456 (56%)	488 (60%)	32 (3.9%)	0.10
MFR ≥ 2 (unitless ratio)	816	543 (67%)	485 (59%)	58 (7.1%)	0.0008
Whole left-ventricle					
Stress MBF ≥ 2.0 mL/min/g or MFR ≥ 2 (unitless ratio)	48	40 (78%)	41 (80%)	1 (2.0%)	0.81
17-Segment level					
Stress MBF ≥ 2.0 mL/min/g or MFR ≥ 2 (unitless ratio)	816	617 (76%)	613 (75%)	2 (0.2%)	0.64

### Analysis of the Three Excluded Patients

**Figure 5:**
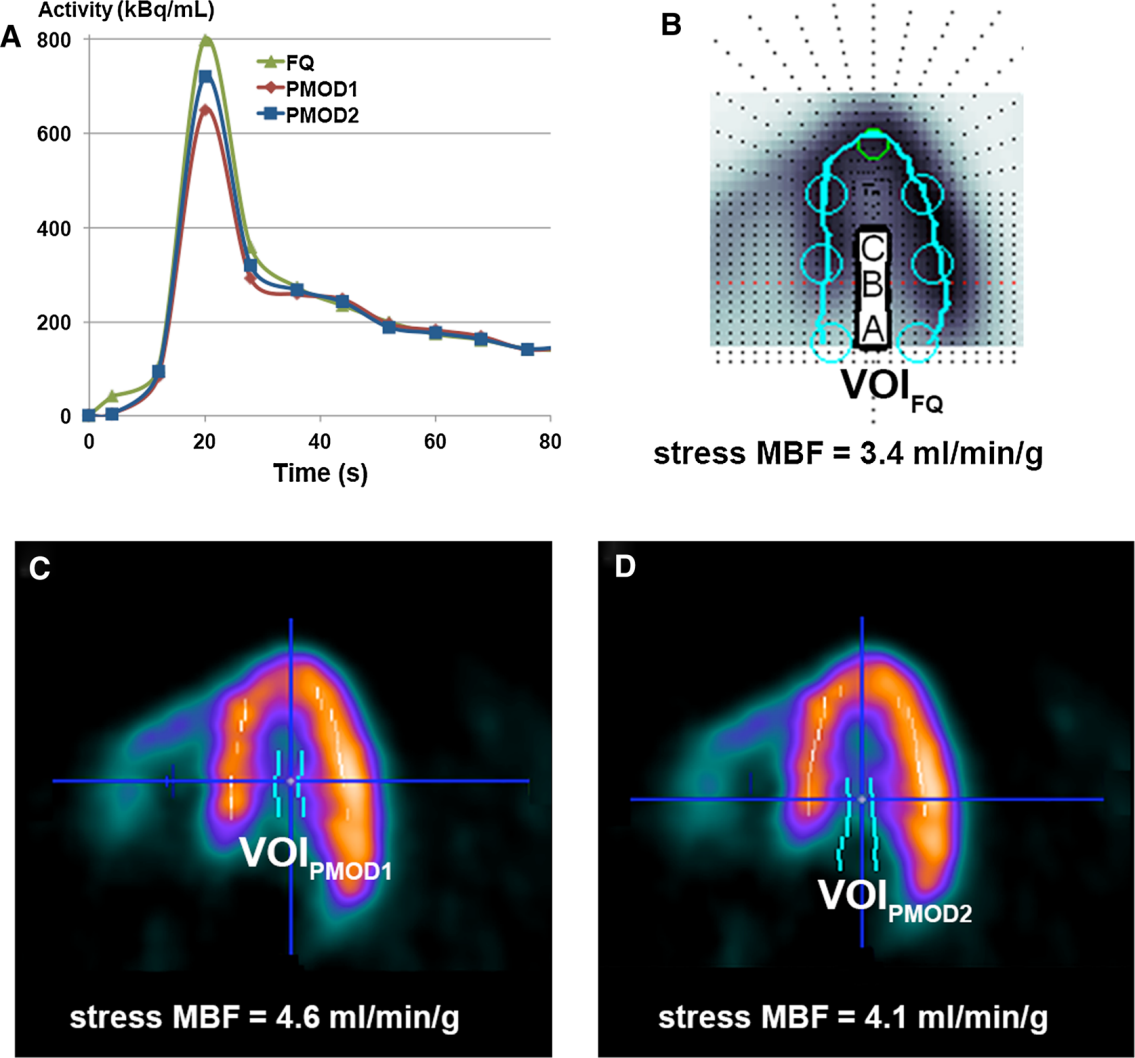
(**A**) Comparison of* left ventricular* blood pool input function (activity in voxel of interest [VOI] vs time) for a patient with differences in size and placement of the VOI leading to differences in stress LV MBF between (**B**) FQ and (**C**) PMOD software packages. When a similar VOI was used in PMOD as in FQ, differences were much smaller (**D**)

Interestingly, the automatic left ventricle VOI delineation was discordant between software packages in 3 subjects at stress. This resulted in a relative overestimation of MBF with PMOD compared to FlowQuant (difference 1.5 ± 0.4 mL/min/g, *P* = .004). For these 3 patients, summed uptake images had the same orientation on both software packages. While left blood spillover values with PMOD remained in the normal range and were not different from FlowQuant values, the *K*_1_ and *k*_2_ values were higher and DV (*K*_1_/*k*_2_) lower with PMOD as compared to FlowQuant. Since the values of the “*a*” and “*b*” parameters of the extraction function were the same for both software packages, as reported by Lortie et al.^14^ we attributed the discrepancy to differences in input functions resulting in overestimation of the *K*_1_ parameter value. Examination of dynamic sequences with PMOD revealed that for two of the three patients, the LV VOI used for the input function was sub-optimally located. In the first patient, the maximal blood input activity was shifted basally. With PMOD, the automatic VOI was placed too deep in the LV cavity leading to underestimation of the input function (Figures 5A, 5C). In contrast, FlowQuant correctly placed and used the median TAC of three atrial, basal, and cavity TACs (Figure 5B). Correction of the PMOD VOI position led to increased blood input function and decreased MBF differences among software packages (Figure 5D). In the third patient, while not detectable on the summed uptake image, examination of the dynamic sequence revealed patient motion during tracer infusion. PMOD’s blood VOI was therefore too deep in the cavity, whereas FlowQuant’s VOI was more posterior, resulting in a more appropriate input function. As for the first patient, manual correction of PMOD’s blood VOI diminished the MBF difference between software methods.

## Discussion

To our knowledge this is the first study comparing two software packages commonly used for MBF quantification with ^82^Rb PET with detailed statistical analysis of concordance. In comparing these two software packages, we found several differences, whereas the LV reorientation process was successfully run with both PMOD and FlowQuant, there was a large difference in automatic VOI determination. FlowQuant processing failed in only 6% of the scans, whereas PMOD VOI determination had to be manually determined for approximately one-third of the studies. Once VOIs were well placed, the rest of the processing ran automatically for both software packages.

Although myocardial uptake images were similar, there were significant differences regarding quantitative results. The overall rest MBF, stress MBF, and MFR assessed from ^82^Rb-PET data were similar to the CAD population of a previous study using the same kinetic model,^14^ with higher rest MBF and, lower stress MBF and MFR as compared to previous publications in healthy volunteer populations.^14^,^16^ Stress global LV MBF was significantly higher using PMOD, leading to slightly higher values of MFR as compared to FlowQuant, but of little clinical relevance, however. We found similar results using global LV MBF or 17-segment MBF, suggesting that differences in MFR could neither be due to differences in reorientation, as qualitatively assessed when looking at uptake image reorientation, nor to myocardial sampling or to two-compartment model characteristics.^11^ Moreover, the absence of significant difference between PMOD and FlowQuant for septal and non-septal segments emphasizes that it cannot be due to differences in RV blood spillover correction in the septum. Thus, our study shows that changes due to RV blood spillover correction methods are not significant, which is contrary to what has been hypothesized as a source of possible difference among software packages recently by Tahari et al.^23^

For three patients with difference in automatic left ventricle VOI delineation, we found that two of the three higher MBF values with PMOD were partly due to lower input functions leading to overestimation of *K*_1_ and stress MBF. This highlights the importance of careful blood pool sampling for the arterial input function, as recently investigated by Vasquez et al^30^ and Armstrong et al,^31^ with increased MBF value in case of decreased arterial input function. The higher MBF values by PMOD as compared to FlowQuant occurred at the highest range of MBF, where small differences in *K*_1_ are amplified by higher extraction correction factors.

Regarding the perfusion status, there was a small but statistically significant difference between homogeneous vs heterogeneous polar maps for stress global LV MBF with FlowQuant (*P* = .009) or with PMOD (*P* = .036). MFR was however significantly lower for patients with heterogeneous perfusion than for patients with homogeneous perfusion using FlowQuant (*P* = .022) but not using PMOD (*P* = .2). Although there were higher values for stress MBF and MFR with PMOD as compared to FlowQuant, the concordance was very good as reflected by Pearson correlation values > 0.83 for global estimates. Moreover, differences mainly concerned stress MBF ≥ 2.0 mL/min/g, which might not result in different clinical management in daily practice. Although we can be confident that both packages may be used interchangeably in the clinical setting from the present study, it cannot be inferred from our data that this would hold true when performing multi-center trials or for studies designed to determine specific MBF or MFR thresholds based on normal populations.^22^,^32^

Although similar studies exist on multiple software package comparisons for MBF quantification,^20^-^24^ including PMOD and FlowQuant,^22^ the present study presents a more detailed comparison using Lin’s concordance analysis, which allows to grasp the nature of the observed difference and shows no systematic bias (agreement cannot be improved by a linear transformation of the results) and that any disagreement was mostly due to measurement precision.

## New Knowledge Gained

The present work comparing two software packages using contemporary statistical methods (analysis of concordance) was able to discriminate between measurement precision and bias to understand the nature of the observed differences. The lack of systematic bias between methods suggests that they may be used interchangeably and shows that observed variations were mostly due to measurement precision. Although group comparison of multiple software programs exist, one-to-one concordance analysis allows better understanding of the disagreements and is needed before data from two software packages can be pooled in multi-center studies or before thresholds derived from one software package can be applied to another.

## Conclusion

While faster and more often successful automatic processing was achieved with FlowQuant, both software packages led to very similar results. Concordance in measured values was excellent for quantification of rest MBF, stress MBF, and MFR without systematic differences, despite relatively higher values with PMOD as compared to FlowQuant, in particular at the highest MBF leading to mild overestimation of MFR. There were no difference between FlowQuant and PMOD regarding the ability to distinguish between normal vs abnormal flow at the whole LV level. A small difference was seen at the segmental level in the number of normal segments, but it is not expected to lead to difference in clinical management. We conclude that both software packages can be used interchangeably for analyzing clinical ^82^Rb dynamic cardiac PET studies in daily practice.

## Abbreviations

BP: Blood pool
FWHM: Full-width half maximum
LOA: Limit of agreement
LV: Left ventricle
MBF: Myocardial blood flow
MFR: Myocardial flow reserve
PET: Positron emission tomography
82Rb: Rubidium-82
TAC: Time-activity curve
VOI: Volume of interest
